# Variability in gene cassette patterns of class 1 and 2 integrons associated with multi drug resistance patterns in *Staphylococcus aureus* clinical isolates in Tehran-Iran

**DOI:** 10.1186/s12866-015-0488-3

**Published:** 2015-07-31

**Authors:** Mahdi Mostafa, Seyed Davar Siadat, Fereshteh Shahcheraghi, Farzam Vaziri, Alireza Japoni-Nejad, Jalil Vand Yousefi, Bahareh Rajaei, Elnaz Harifi Mood, Nayyereh Ebrahim zadeh, Arfa Moshiri, Seyed Alireza Seyed Siamdoust, Mohamad Rahbar

**Affiliations:** Department of Mycobacteriology & Pulmonary Research, Pasteur Institute of Iran, Tehran-Iran, No. 358, 12th Farwardin Ave, Jomhhoori St, Tehran, 1316943551 Iran; Department of Microbiology, Karaj branch, Islamic Azad University, Karaj, Iran; Department of Microbiology, Pasteur Institute of Iran, Tehran, Iran; National Institute of Genetic Engineering and Biotechnology, Tehran, Iran; Biotechnology Department, Faculty of Medicine, Shahid Beheshti University of Medical Sciences, Tehran, Iran; Department of Anesthesiology, Iran University of Medical Sciences, Tehran, Iran; Department of Microbiology, Reference Health Laboratories Research Center, Ministry of Health and Medical Education, Tehran, Iran

**Keywords:** *Staphylococcus aureus*, Gene cassettes, Integrons, Multidrug-resistant, Iran

## Abstract

**Background:**

To investigate antibiotic resistance, the occurrence and distribution of class 1 and 2 integrons in multidrug- resistant *Staphylococcus aureus* isolates from hospitals in Tehran, Iran.

The isolates were examined for susceptibility to antimicrobial agents. The *mecA* gene, class 1 and 2 integrons were detected by PCR. Integrase positive strains were further analysed for the presence of resistance gene cassettes using specific primers and were sequenced.

**Results:**

Among 139 *S.aureus* isolates, 109 (78.4 %) and 112 (80.5 %) strains were considered as multidrug resistant and *mecA* positive, respectively. Class 1 integrons and internal variable regions were found in 72.6 % (101/139) and 97 % (98/101) and class 2 integrons and variable regions also in 35.2 % (49/139) and 65.3 % (32/49) of *S.aureus* clinical isolates, respectively. Twelve distinct cassette arrays were found, containing genes encoding resistance to β-lactams, aminoglycosides, streptothricin, trimethoprim, chloramphenicol,a putative glucose dehydrogenase precursor and a protein with unknown function. Gene cassette arrays *aadB*, *aadA2* and *dhfrA1-sat2-aadA1* were common in *S.aureus* isolates. We detected a completely new gene cassettes which contained *aadB*, *oxa2*, *aacA4*, *orfD-aacA4-catB8*, *aadB-catB3*, *orfD-aacA4* and *aadB-aadA1-cmlA6* of class 1 and *dhfrA1-sat2-aadA1*, *dhfrA11*, *dhfrA1-sat2* of class 2 integrons.

**Conclusions:**

This is the first study to report carriage of class 1 and 2 integrons and associated gene cassettes among in *S.aureus* isolates from Iran.

## Background

*Staphylococcus aureus* has long been recognized as a major human pathogen responsible for a wide range of infections, from mild skin infections to wound infections and bacteraemia [1]. Although the introduction of antibiotics has lowered the mortality rate from *S. aureus* infections, the bacteria have developed resistance mechanisms to all antimicrobial agents that have been produced [1].

Despite antibiotic therapy, staphylococcal infections occur regularly in hospitalized patients and have severe consequencess [2, 3]. Due to an increasing number of infections caused by methicillin-resistant *S. aureus* (MRSA) strains, which are now most often multiresistant, therapy has become problematic [4]. Since this organism can spread easily by direct or indirect contact between patients and the environment, or among patients and medical personnel, *S.* aureus is an important cause of nosocomial infection, and major outbreaks are common [5, 6].

Dissemination of antibiotic resistance genes by horizontal gene transfer has led to the rapid emergence of antibiotic resistance among bacteria, thus complicating the treatment of infections [7]. Acquired resistance evolves via horizontal transfer of antimicrobial resistance genes located on various types of mobile DNA elements. A key system involved in spreading antibiotic multiresistance is the integron, an element that, although normally immobile itself, can be transferred through mobile genetic elements [8, 9]. Integrons are genetic elements that incorporate exogenous open reading frames by recombination and convert them to functional genes [10]. All integrons characterized to date are composed of these major elements: a gene (*intI*) encoding an integrase; a primary recombination site (*attI*); and an outward orientated promoter (Pc) that directs transcription of the captured genes [11].

In this study, the presence of integrons and gene cassettes in *S. aureus* has been studied for the first time in Iran.

The objective of this research was to identified multiple drug resistance in clinical isolates of *s. aureus* by antibiotic sensitivity testing, detection of *mecA* gene for methicillin-resistant *S. aureus* strains, and distribution of class 1 and 2 integrons and associated gene cassettes among *S.aureus* isolates collected from clinical sources in Tehran by PCR assays and investigates associations between multidrug resistance and the existence of integrons.

## Methods

### Sampling and bacterial isolation

One hundred and thirty nine *S. aureus* isolates were collected from hospitals in Tehran, over a six month period, starting from the first of October 2011 till the end of March 2012 . The isolates were recovered from wounds (*n* = 31), blood (*n* = 31), urines (*n* = 28), nasal swabs (*n* = 21), catheters (*n* = 5), sternum samples (*n* = 5), sputa (*n* = 4), eyes (*n* = 2), CSF (*n* = 1), synovial fluid (*n* = 1), throat samples (*n* = 3) and abscesses (*n* = 7). The isolates were identified as *S. aureus* by standard biochemical tests for catalase, coagulase, clumping factor, DNase, and thermostable nuclease. All isolates were also evaluated for the presence of the sa442 gene by PCR [3, 12]. The research was approved by the Ethics committee of the Pasteur Institute of Iran (no = 402) and acquire either verbal or written informed consent from the patients to take their samples for this study.

### Antibiotic susceptibility test

The isolated S. aureus strains were tested for their *in vitro* antimicrobial susceptibility using the disk diffusion technique on Mueller- Hinton agar (Mast, UK). The results were recorded after 18 h of incubation at 37єC. Antimicrobial drugs tested included Penicillin G; PG(30 μg), Amoxicillin; A(25 μg), Linezolid; LZD(30 μg), Streptomycine; S(10 μg), Ampicillin; AP(10 μg), Tetracycline; T(30 μg), Chloramphenicol; C (30 μg), Sulfamethoxazole-Trimethoprim; SXT(30 μg), Amikacin; AK(30 μg), Azithromycin; ATH(15 μg), Spectinomycin; SPC(100 μg), Gentamicin; GM(10 μg), Vancomycin; VA(30 μg), Teicoplanin; TEC(30 μg), Ciprofloxacin; CIP(5 μg), Oxacillin; OX(1 μg), Levofloxacin; LEV(5 μg), Gatifloxacin; GAT(5 μg), Rifampin; RP(5 μg), Erythromycin; E(15 μg), Ceftriaxone; CRO(30 μg), Tobramycin; TN(10 μg), Clindamycin; CD(2 μg) . The MIC to vancomycin was determined by Etest (bioMeґrieux). Enterococcus faecalis ATCC 29212 and Enterococcus faecium BM4147 were used as controls for vancomycin susceptibility and vancomycin resistance, respectively. The breakpoints for antibiotic susceptibility were determined according to the guidelines of the Clinical and Laboratory Standards Institute (CLSI) [13]. *E. coli* ATCC 25922 was used as a reference control strain. Isolates of S.aureus that show resistance to three or more than three classes of antibiotics, considered as multidrug – resistant strains.

### The extraction of *S. aureus* plasmid and genomic DNA

The genomic DNA preparation used in this study was performed following Sambrook et al. [14]. The DNA was extracted with phenol-chloroform and precipitated by ice-cold ethanol. The DNA pellet was washed with 70 % ethanol and resuspended in 50 μl of TE buffer (10 mM Tris; 1 mM EDTA, pH8) and stored at -20єC until used and 1 μl of the suspension were used as the template DNA for PCR. Using the primers determined [3] *mecA* gene was detected by PCR. Size of the amplified fragment was 533 bp.

The Qiagen Plasmid Midi Kit was used for the *S. aureus* plasmid DNA extraction; the manufacturer’s instructions were followed.

### Detection of class 1 and 2 integrons

All isolates were screened for detection of class 1 and 2 integrons by the primers described by Moura et al. [15] designed for the *intI1* and *intI2* genes respectively. Primers intI1 F/R and intI2 F/R were used to amplify 280 bp and 232 bp fragments, respectively. The amplification program was performed by termocycler (Eppendorf Mastercycler®, MA) and started with initial denaturation of 4 min at 94 °C and programmed wi th 35 cycles of each: 1 min at 94 °C, 30 s at 55 °C, 1 min at 72 °C. The program finished with the final extension of 10 min at 72 °C.

### Mapping of class 1 and 2 integrons

The gene cassettes inserted in the variable regions of class 1 and 2 integrons (*IVR*) were amplified using the primer pairs introduced by Moura et al. [15].

### Sequencing of amplified integron gene cassettes

For each PCR product that had a unique size when visualized on the gel, a number of samples of the post-PCR mixture was processed with the High Pure PCR Product Purification kit (Roche,USA) and used for direct sequencing. The purified amplicons were sequenced using the ABI Capillary System (Pasteur Institute, Tehran, Iran). Resulting sequences were assembled by using SeqMan program within the Lasergene suite version 7 (DNAstar Inc, Madison, WI, USA). Potential open reading frames (ORFs) were predicted by using the NCBI (National Center for Biotechnology Information) ORF Finder tool (http://www.ncbi.nlm.nih.gov/gorf/gorf.html). BLAST (http://blast.ncbi.nlm.nih.gov/Blast.cgi) against GenBank database and The Integron Database INTEGRALL (http://integrall.bio.ua.pt/) were performed repeatedly for sequence comparison and annotation.

### Statistical analysis

The antibiotic resistance data between integron-positive isolates and integron-negative isolates was compared and the *χ*2 test was used to calculate the *P* value in terms of resistant and susceptible numbers by SPSS version 18 software. A *P* value of <0.05 was considered statistically significant.

## Results

A total of 139 *S. aureus* isolates were collected from clinical specimens of hospitals in Tehran, over a 6 month period, starting from the first of October 2011 till the end of March 2012.

### Antibiotic susceptibility test

**Fig. 1:**
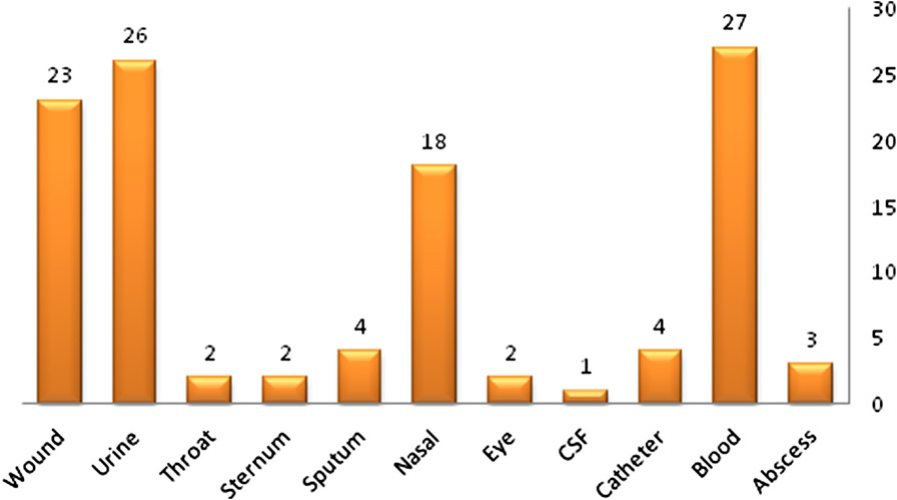
Distribution of *mecA* gene in *MRSA* isolates from nosocomial sources

The antimicrobial resistance patterns of isolates are shown in Tables 1 and 2. According to the results of the vancomycin Etest, all isolates were susceptible to this agent. Of the 139 isolates, 109 (78.4 %) isolates were designated as MDR *S.aureus*. Overall, 112 (80.5 %) isolates were resistant to methicillin and were confirmed as MRSA based on the detection of the mecA gene. Distribution of *mecA* gene in *MRSA* isolates from nosocomial sources are shown in Fig. 1. All of the MRSA isolates were susceptible to Vancomycin and Teicoplanin (100 %). Also all MRSA isolates were resistant to Amoxicillin (100 %) and Ampicillin (100 %), Tetracycline 69 (61.6 %), Amikacin 40 (35.7 %), Ceftriaxone 68 (60.7 %), Gentamicin 73 (65.1 %), Spectinomycin 77 (68.7 %), Ciprofloxacin 51 (45.5 %), Chloramphenicol 45 (40.1 %), Oxacillin 93 (83 %), Gatifloxacin 39 (34.8 %), Erythromycin 89 (79.4 %), Azithromycin 67 (59.8 %), Tobramycin 57 (50.8 %), Levofloxacin 55 (49.1 %), Rifampin 37 (33 %), Penicillin G 111 (99.1 %), Streptomycin 69 (61.6 %) and Clindamycin 73 (65.1 %). The least resistance to the following antibiotics are observed: Sulfamethoxazole-Trimethoprim 34 (30.3 %), and Linezolid 18 (16 %).

**Table 1:** Correlation of antibiotic resistance between class 1 integron-positive and integron-negative of *S.aureus*

Antibiotics	All strains of S.aureus (*n* = 139)		Integron-positive isolates (*n* = 101)		Integron-negative isolates (*n* = 38)		*P* value
	R	S	R	S	R	S	
AK*	43 (31 %)	96 (69 %)	35 (34.7 %)	66 (25.3 %)	8 (21 %)	30 (79 %)	0.088
SXT*	38 (27.3 %)	101 (72.7 %)	34 (33.7 %)	67 (66.3 %)	4 (10.5 %)	34 (89.5 %)	0.004
C*	49 (32.3 %)	90 (64.7 %)	41 (40.6 %)	60 (59.4 %)	8 (21 %)	30 (79 %)	0.024
TE*	78 (56.1 %)	61 (43.9 %)	65 (64.4 %)	36 (35.6 %)	13 (34.2 %)	25 (65.8 %)	0.001
CIP*	56 (40.3 %)	83 (59.7 %)	49 (48.5 %)	52 (51.5 %)	7 (18.4 %)	31 (81.6 %)	0.001
GM*	76 (54.7 %)	63 (45.3 %)	68 (67.3 %)	33 (32.7 %)	8 (21 %)	30 (79 %)	<0.001
SPC*	86 (61.9 %)	53 (38.1 %)	73 (72.3 %)	28 (27.7 %)	13 (34.2 %)	25 (65.8 %)	<0.001
ATH*	69 (49.6 %)	70 (50.4 %)	62 (61.4 %)	39 (38.6 %)	7 (18.4 %)	31 (81.6 %)	<0.001
S*	75 (54 %)	64 (46 %)	61 (60.4 %)	40 (39.6 %)	14 (37 %)	24 (63 %)	0.011
GAT	44 (31.7 %)	95 (68.3 %)	32 (31.7 %)	69 (68.3 %)	12 (31.6 %)	26 (68.4 %)	0.581
OXA	104 (74.8 %)	35 (25.2 %)	76 (75.2 %)	25 (24.8 %)	28 (73.7 %)	10 (26.3 %)	0.505
PG	137 (98.6 %)	2 (1.4 %)	101 (100 %)	0 (0 %)	36 (94.7 %)	2 (5.3 %)	0.073
RP	43 (31 %)	96 (69 %)	31 (30.7 %)	70 (69.3 %)	12 (31.6 %)	26 (68.4)	0.537
LZD	24 (17.3 %)	115 (82.7 %)	20(19.8 %)	81 (80.2 %)	4 (10.5 %)	34 (89.5 %)	0.149
CD	84 (60.4 %)	55 (39.6 %)	65 (64.4 %)	36 (35.6 %)	19 (50 %)	19 (50 %)	0.089
E*	95 (68.3 %)	44 (31.7 %)	78 (77.2 %)	23(22.8 %)	17 (44.7 %)	21 (55.3 %)	<0.001
TN	66 (47.5 %)	73 (52.5 %)	46 (45.5 %)	55 (54.5 %)	20 (52.6 %)	18 (47.4 %)	0.289
LEV*	56 (40.3 %)	83 (59.7 %)	49 (48.5 %)	52 (51.5 %)	7 (18.7 %)	31 (81.6 %)	0.001
CRO*	71 (51.1 %)	68 (48.9 %)	66 (65.3 %)	35 (34.7 %)	5 (13.1 %)	33 (86.9 %)	<0.001

*: significant values, *AK* Amikacin, *SXT* Trimethoprim- Sulfamethoxazole, *C* Chloramphenicol, *TE* Tetracycline, *CIP* Ciprofloxacin, *GM* Gentamicin, *SPC* Spectinomycin, *ATH* Azithromycine, *S* Streptomycin, *GAT* Gatifloxacin, *OXA* Oxacillin, *PG* Penicillin G, *RP* Rifampin, *LZD* Linezolid, *CD* Clindamycin, *E* Erythromycin, *TN* Tobramycin, *LEV* Levofloxacin, *CRO* Ceftriaxone; *R* Resistant, *S* sensitive

**Table 2:** Correlation of antibiotic resistance between class 2 integron-positive and integron-negative of *S. aureus*

Antibiotics	All strains of S.aureus (*n* = 139)		Integron-positive isolates (*n* = 49)		Integron-negative isolates (*n* = 90)		*P* value
	R	S	R	S	R	S	
AK*	43 (31 %)	96 (69 %)	21 (42.9 %)	28 (57.1 %)	22 (24.4 %)	68 (75.6 %)	0.021
SXT*	38 (27.3 %)	101 (72.7 %)	24 (49 %)	25 (51 %)	14 (15.5 %)	76 (84.5 %)	<0.001
C	21 (42.9 %)	28 (57.1 %)	21 (42.9 %)	28 (57.1 %)	28 (31.1 %)	62 (68.9 %)	0.116
TE*	78 (56.1 %)	61 (43.9 %)	38 (77.5 %)	11 (22.5 %)	40 (44.4 %)	50 (55.6 %)	<0.001
CIP*	56 (40.3 %)	83 (59.7 %)	28 (57.1 %)	21 (42.9 %)	28 (31.1 %)	62 (68.9 %)	0.003
GM*	76 (54.7 %)	63 (45.3 %)	37 (75.5 %)	12 (24.5 %)	39 (43.3 %)	51 (56.7 %)	<0.001
SPC*	86 (61.9 %)	53 (38.1 %)	41 (83.7 %)	8 (16.3 %)	45 (50 %)	45 (50 %)	<0.001
ATH*	69 (49.6 %)	70 (50.4 %)	36 (73.5 %)	13 (26.5 %)	33 (36.7 %)	57 (63.3 %)	<0.001
S*	75 (54 %)	64 (46 %)	34 (69.4 %)	15 (30.6 %)	41 (45.6 %)	49 (54.4 %)	0.006
GAT	44 (31.7 %)	95 (68.3 %)	19 (38.8 %)	30 (61.2 %)	25 (27.8 %)	65 (72.2 %)	0.127
OXA	104 (74.8 %)	35 (25.2 %)	41 (83.7 %)	8 (16.3 %)	63 (70 %)	27 (30 %)	0.056
PG	137 (98.6 %)	2 (1.4 %)	49 (100 %)	0 (0 %)	88 (97.8 %)	2 (2.2 %)	0.418
RP	43 (31 %)	96 (69 %)	18 (36.7 %)	31 (63.3 %)	25 (27.8 %)	65 (72.2)	0.184
LZD	24 (17.3 %)	115 (82.7 %)	9 (18.4 %)	40 (81.6 %)	15 (16.7 %)	75 (83.3 %)	0.486
CD	84 (60.4 %)	55 (39.6 %)	34 (69.4 %)	15 (30.6 %)	50 (55.6 %)	40 (44.4 %)	0.078
E*	95 (68.3 %)	44 (31.7 %)	40 (81.6 %)	9 (18.4 %)	55 (61.1 %)	35 (38.9 %)	0.01
TN	66 (47.5 %)	73 (52.5 %)	26 (53 %)	23 (47 %)	40 (44.4 %)	50 (55.6 %)	0.214
LEV*	56 (40.3 %)	83 (59.7 %)	28 (57.1 %)	21 (42.9 %)	28 (31.1 %)	62 (68.9 %)	0.003
CRO*	71 (51.1 %)	68 (48.9 %)	38 (77.5 %)	11 (22.5 %)	33 (36.7 %)	57 (63.3 %)	<0.001

*: significant values *AK*, Amikacin, *SXT* Trimethoprim- Sulfamethoxazole, *C* Chloramphenicol, *TE* Tetracycline, *CIP* Ciprofloxacin, *GM* Gentamicin, *SPC* Spectinomycin, *ATH* Azithromycine, *S* Streptomycin, *GAT* Gatifloxacin, *OXA* Oxacillin, *PG* Penicillin G, *RP* Rifampin, *LZD* Linezolid, *CD* Clindamycin, *E* Erythromycin, *TN* Tobramycin, *LEV* Levofloxacin, *CRO* Ceftriaxone, *R* Resistant, *S* sensitive

### Detection of class 1 and 2 Integrons

One hundred and thirty-nine of *S.aureus*, were examined for the presence of known markers for integrons by PCR amplification. The 101 (72.6 %) isolates were positive for class 1 integron with the amplicon size of 280 bp and class 2 integron was found in 49 (35.2 %) isolates with the amplicon size of 232 bp. Information about integron positive isolates among different sources of *S.aureus* isolates are shown in Table 3. In the present study, among of 112 strains of MRSA, 95 (84.8 %) isolates carrying class 1 integron as well as 46 (41 %) isolates carrying class 2 integron.

**Table 3:** Information about integron positive isolates among different sources of *S.aureus* isolates

Isolation source(No)	No. of intI1 positive isolates (%)	No. of intI2 positive isolates (%)	No. of intI1 and int2 positive isolates (%)
Abscesses (7)	4	3	3
Blood (31)	24	11	10
Catheter (5)	3	3	3
CSF (1)	1	0	0
Eye (2)	2	0	0
Nasal (21)	14	1	1
Sputum (4)	2	3	2
Sternum (5)	2	0	0
Throat (3)	2	3	2
Urine (28)	25	11	11
Wound (31)	22	14	12
Synovia (1)	0	0	0
Total (139)	101 (72.6 %)	49 (35.2 %)	44 (31.6 %)

The results indicate that resistance to several different antibiotics is associated with the presence of the integron. Tables 1 and 2 shows the antibiotic susceptibilities of integron-positive and integron-negative isolates to each of the antibiotics. Both class 1 and 2 integrases genes were present together in 44 (31.6 %) strains of *S.aureus*.

Antimicrobial susceptibility testing showed the multidrug resistance rates of integron-positive class 1 and negative strains were 72.6 % (101/139) and 27.4 % (38/139) and also rates of integron-positive class 2 and negative strains were 35.2 % (49/139) and 64.8 % (90/139), respectively.

Of 101 isolates carrying class 1 integrons, 89 (88.1 %) isolates amplified class 1 integrons on chromosome, 12 (11.9 %) isolates on plasmid. Among 49 isolates harbored class 2 integrons, 11 (22.4 %) isolates carried class 2 integrons only on chromosome and 38 (77.6 %) isolates on plasmid.

### Characterization of gene cassettes in class 1 and 2 integrons

**Fig. 2:**
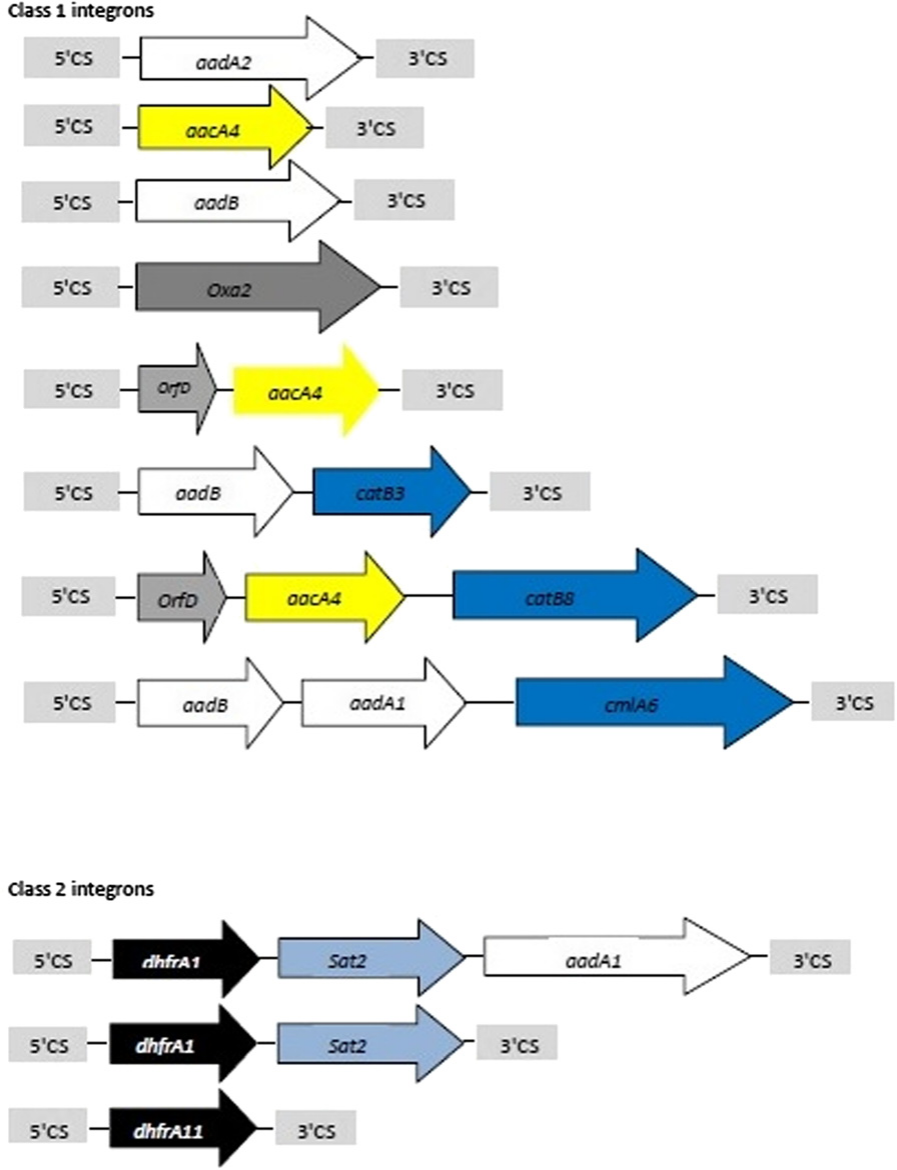
Schematic representation of the various cassette arrays found in class 1 and class 2 integrons. The arrows display the open reading frames of the different genes. All *aadA* and *aadB* genes are presented as white arrows, *dhfrA*, *aacA4*, *orfD, oxa2, catB, cmlA6, sat2* genes are presented as black, yellow, light grey, grey, blue and light blue arrows respectively. The grey light boxes indicate the 3′ and 5′ CSs of class1 or class 2 integrons

Of the 139 s.aureus isolates, 101 (72.6 %) isolates were identified as being positive for class 1 integron. PCR amplification of the integron cassette region occurred in 98 (97 %) class 1 integron containing isolates (Table 4). Class 2 integron was detected in 49 (35.2 %) isolates. The integron cassette region could not be amplified by PCR in 17 (34.6 %) of the class 2 integron-containing isolates (Table 5). Schematic representation of the various cassette arrays found in class 1 and class 2 integrons are shown in Fig. 2.

**Table 4:** Sizes of variable regions of integron class I cassettes in intI1 positive isolates

Pattern of integron I cassettes bands (bp)	No. of isolates (%)
500	1 (0.99)
750	49 (48.5)
800	3 (2.9)
1000	36 (35.6)
1100	1 (0.99)
1200	3 (2.9)
1420	2 (1.98)
2120	1 (0.99)
3110	2 (1.98)
Without PCR product	3 (2.9)
Total no. of intI1 positive isolates	101 (100)

**Table 5:** Sizes of variable regions of integron class 2 cassettes inintI2 positive isolates

Pattern of integronII cassettes bands (bp)	No. of isolates (%)
750	8 (16.3)
1238	5 (10.2)
2500	19 (38.7)
Without PCR product	17 (34.6)
Total no. of intI2 positive isolates	49 (100)

### Nucleotide sequence accession numbers

The nucleotide sequence data reported in this article are available in the GenBank nucleotide database under GenBank accession numbers KF030468, KF411134, KF305706, KF411137, KF411135, KF411133, KF356395, KF305707, KJ002505, KJ002506,KF015994,KF015995,KF411138,KF411139 obtained from the gene cassette of class 1 integrons and KF305708, KF305710, KF411136, KJ769139 obtained from the gene cassette of class 2 integrons, overall, 18 strains were sequenced (Table 6).

**Table 6:** Phenotypic and genotypic characterization of isolates by different length of amplicons

Strain	Source	Size of 5′CS-3′CS amplicons	Intl gene	Gene cassette array	Integron putative location^a^	Resistance phenotype	Accession number
Sm59	wound	1200 bp	I	blaoxa2	C	CRO,LEV,TN,E,CD,LZD,RP,PG,OXA,A,S,ATH,GM,CIP,AP,SXT	KF356395
MMS1	wound	750 bp	I	aadB	C	CRO,E,CD,PG,A,S,ATHSPC,GM,CIP,AP,TE,C,AK	KF015994
s61	wound	750 bp	I	aadB	C	CRO,TN,E,CD,PG,OXA,A,S,SPC,GM,CIP,AP,TE,SXT	KF030468
sm54	blood	1200 bp	I	blaoxa2	C	CRO,LEV,TN,E,CD,LZD,RP,PG,OXA,A,S,ATH,GM,CIP,AP,SXT	KF305707
sa10	blood	800 bp	I	aacA4	C	CRO,LEV,E,CD,LZD,RP,PG,OXA,A,S,ATH,CIP,AP,TE,SXT	KF305706
S232	blood	500 bp	I	putative glucose dehydrogenase-hypothetical protein	C	CRO,E,CD,PG,OXA,A,S,SPC,AP,TE	KF411133
MMS2	abscess	750 bp	I	aadB	C	CRO,E,CD,PG,A,S,ATH,SPC,GM,CIP,AP,TE,C,AK	KF015995
s74	blood	1000 bp	I	aadA2	C	CRO,TN,E,CD,PG,OXA,A,S,SPC,GM,CIP,AP,TE,SXT	KF411134
s54	blood	2120 bp	I	orfD-aacA4-catBB	P	CRO,LEV,E,CD,LZD,PG,OXA,A,S,ATH,SPC,GM,CIP,AP,TE,C,AK	KF411135
s5	wound	1100 bp	I	orfD-aacA4	C	CRO,LEV,E,CD,LZD,PG,OXA,A,S,ATH,SPC,GM,CIP,AP,TE,C,AK	KF411137
s22	wound	1000 bp	I	aadA2	C	CRO,TN,E,CD,PG,OXA,A,S,SPC,GM,CIP,AP,TE,SXT,AK	KF411138
s36	blood	1000 bp	I	aadA2	C	CRO,TN,E,CD,PG,OXA,A,S,SPC,GM,CIP,AP,TE,SXT	KF411139
sm91	blood	1420 bp	I	aadB-catB3	C	CRO,TN,E,CD,PG,OXA,A,S,SPC,GM,APC,SXT	KJ002505
sm105	wound	3100 bp	I	aadB-aadA1-cmlA6	P	CRO,LEV,E,CD,LZD,PG,OXA,A,S,ATH,SPC,GM,CIP,AP,TE,C,SXT,AK	KJ002506
sm22	wound	750 bp	II	hdfrA11	C	CRO,LEV,TN,E,CD,PG,OXA,A,S,SPC,GM,CIP,AP,TE,SXT	KF305708
sm97	blood	2500 bp	II	dhfrA1-sat2-aadA1	P	CRO,LEV,TN,E,CD,PG,OXA,GAT,A,S,ATH,SPC,GM,CIP,AP,TE,C,SXT,AK	KF305710
s92	blood	750 bp	II	hypothetical protein	C	CRO,E,CD,PG,OXA,AS,SPC,AP,TE	KF411136
s105	wound	1230 bp	II	dhfrA1-sat2	C	CRO,LEV,TN,E,CD,LZD,RP,PG,OXA,A,S,ATH,GM,AP,SXT	KJ769139

^a^ *c* chromosomal location, *P* plasmid location, *CRO* ceftriaxone, *LEV* levofloxacin, *E* erythromycin, *CD* clindamycin, *TN* tobramycin, *PG* penicillin G, *LZD* linezolid, *OXA* oxacillin, *A* amoxicillin, *S* streptomycin, *ATH* azithromycine, *SPC* spectinomycin, *GM* gentamycin, *CIP* ciprofloxacin, *AP* ampicillin, *TE* tetracycline, *C* chloramphenicol, *SXT* trimethoprim-sulfamethoxazole, *AK* amikacin

## Discussion

In the current study, prevalence of *intI 1* and *intI 2* were examined among multidrug resistant *S.aureus* strains, isolated from different specimens. The prevalence of antimicrobial-resistant bacterial pathogens has become a major public health concern [16, 17]. Horizontal gene transfer has already been proved a significant mechanism for disseminating antimicrobial resistance in bacterial populations. Antimicrobial drug resistance can also be facilitated by integrons in case of many other bacteria [18–21]. Since the integrons were detected first in Gram-negative bacteria, many studies have been conducted on Gram-negative bacteria. Although the role of class 1integrons is well known in the spread of antibiotic resistance genes in gram-negative bacteria, much less is known about gram-positive bacteria and very few studies have reported the presence of class 1 integrons in gram-positive bacteria so increasing antibiotic resistance mediated by integrons in gram-positive bacteria has become a great concern in the medical field [22].

Integrons are genetic elements able to recognize and capture mobile gene cassettes carrying antibiotic resistance genes leading to MDR distribution and subsequently limitation of treatment options for infections [23].

To our knowledge, this is the first study to report carriage of class 1 and 2 integrons and associated gene cassettes in *S.aureus* isolates from Iran. Our findings demonstrate that integrons are widespread among *S.aureus* clinical isolates in Tehran. Most resistant pattern was observed in amoxicillin, ampicillin, oxacillin, erythromycin, tetracycline, gentamicin, tobramycin, streptomycine, spectinomycin, ciprofloxacin, azithromycine, penicillin G, clindamycin, levofloxacin, ceftriaxone and antibiotic such as vancomycin, teicoplanin, linezolid and trimethoprim-sulfamethoxazole were considered as the most effective drugs against *S.aureus* strains that this results largely agrees to findings of Xu et al. about amoxicillin/clavulanic acid, ciprofloxacin, clindamycin, erythromycin, gentamicin, levofloxacin, oxacillin, tetracycline, trimethoprim-sulfamethoxazole and the result of Ren et al. about oxacillin, erythromycin, azithromycin, clindamycin, amoxicillin/clavulanic acid, ciprofloxacin, tetracycline, gentamycin [24, 25].

Among different classes of integrons, class 1 integrons have been found more frequently in different species of bacteria, especially Gram-negative bacteria [11, 26, 27]. The classification of different integrons is mainly based on differences in the gene structure of integrases [11].

In this survey, presence of integrons in strains of *S.aureus* as compared to similar studies conducted by Xu et al. and Ren et al. has been increased [24, 25]. The rate of detection of integron class 1 was more than integron class 2. One hundred and one (72.6 %) isolates were positive for class 1 integron and 49 (35.2 %) isolates were positive for class 2 integron.

The nine different cassettes arrangements wich class 1 integrons and another three arrangements within class 2 integrons were identified.

Twelve different gene cassettes were detected. Cassette genes encoding resistance to aminoglycosides were found to be predominant in the class 1 integron. The *aadB* and *aadA* types genes that encode resistance to aminoglycosides (*aadB* gene cassette conferring resistance to gentamicin, tobramycin, and kanamycin; *aadA* gene cassette conferring resistance to streptomycin and spectinomycin) were most commonly found [11].

The B-lactamase cassettes (*oxa2* or *blaoxa2*) that confer resistance to oxacillin and ampicillin [28], were found in three isolates with amplicon size 1200 bp. The *aacA4* cassette (aminoglycoside acetyl transferase) that confer resistance to gentamicin, amikacin and tobramycin, was found with amplicon size 800 bp [11, 28].

The *dfr* cassettes (*dfrA1*,-*A11*) that confer resistance to trimethoprim, were also detected frequently. The *sat2* cassette confers resistance to streptothricin by encoding a streptothricin acetyltransferase [28, 29].

The diversity of gene cassettes inserted in *IVRs* of class 2 integrons is much lower than class 1 integrons. This reduction in diversity is probably owing to a nonsense mutation in codon 179 (ochre 179) in the integrase gene of class 2 integrons, thereby yielding a truncated, non-functional protein, the resultant integrase is therefore unable to excise existing cassettes or insert new ones [10].

Phenotypic and genotypic characterizations of integrase-positive *S.aureus* are shown in Table 6. Ninety percent (91 out of 101) of class 1 and 8.16 % (4 out of 49) of class 2 integrons harboured at least one gene cassette. Eleven arrays were detected containing one to three cassettes, out of which eight distinct arrays were present in integrons of class 1 and three arrays in class 2 integrons. Common gene cassettes arrays were identified in *S.aureus* strains. In comparison with other sequences deposited in databases, the following gene cassettes were identified: gentamicin, tobramycin and kanamycin resistance genes (*aadB*), gentamicin, amikacin and tobramycin resistance genes (*aacA4*), streptomycin and spectinomycin resistance genes (*aadA1*, *aadA2*), trimethprim resistance genes (*dfrA1*, *dfrA11*), β-lactam resistance genes (*blaoxa2*), chloramphenicol resistance genes (*catB3*, *catB8* and *cmlA6*), streptothricin resistance genes (*sat2*), an *orfD* encoding a protein with unknown function, a putative glucose dehydrogenase. Moreover, the arrays *aadB* and *aadA2* in class 1 integrons were found during the entire process, indicating the most common array. Previous studies have reported *aadA2*, *aacA4-cmlA1*, *dfrA17-aadA5* and *dfrA12*-*orfF*-*aadA2* genes as frequently found gene cassettes both in clinical and environmental strains [30, 31].

Common gene cassette arrangements in class 2 integrons were found in *S.aureus* strains (*dfrA1*-*sat2*-*aadA1*). It should be noted, the cassette array *dfrA1-sat2-aadA1* was detected most frequently in class 2 integrons [15, 30–32]. Furthermore, integron-positive isolates were resistant to different classes of antimicrobial agents but related resistance gene cassettes were not found to be harbored on the integron, implying the nonintegron sources of resistance to these antibiotics [33]. In this study, integron cassette region could not be amplified by PCR in 3 (2.9 %) and 17 (34.6 %) of the class 1 and 2 integron-containing isolates respectively. In the cases where the cassette array could not be amplified, there is no cassettes or in the absent, significant alter-ation in the 3 CS primer binding site or a very large size of the Integron cassette are the most likely explanations for the negative PCRs [3].

The total prevalence of integrases carried in plasmids 36 % (50/139) was similar to that previously reported in *S.aureus* [34]. Nevertheless, we are aware that the prevalence obtained may be biased due to the fact that plasmid and chromosomal DNA can display the same electrophoretic mobility. The dissemination of the class 1 integrons, in many instances, has been attributed to the spread of an integron-containing transposon, Tn*21* [35]. The distribution of Tn*7* in clinical isolates correlates with the frequency of trimethoprim resistance [36]. Trimethoprim resistance is due to the dihydrofolate reductase enzyme encoded by the *dhfr* gene in Tn*7* [29]. Class 2 integron variable regions were amplified using primers that bind to attI2 and to orfX, situated downstream of the cassette region within transposon Tn7 [37]. Therefore, the presence of such an integron in *S.aureus* may have had its origin in a transposition event.

Antimicrobial resistance patterns revealed that 78.4 % of the integrase-positive strains were multiresistant (i.e. resistant to 3 or more antibiotics).

## Conclusions

This is the first report of the presence of classes 1 and 2 integrons in isolates of *S.aureus in Iran*. These results indicated with the spread of MDR strains, class 1 and 2 integrons carrying gene cassettes are widely disseminated among *S.aureus* strains in our hospital. In cases that significant relationship between the presence of integrons and antibiotic resistance were not observed, resistance could be achieved by different ways such as deficiency in cell wall enzymes or resistance under plasmid or chromosome control [38].
